# Comparison of the effect of double-lumen endotracheal tubes and bronchial blockers on lung collapse in video-assisted thoracoscopic surgery: a systematic review and meta-analysis

**DOI:** 10.1186/s12871-022-01876-2

**Published:** 2022-10-29

**Authors:** Ying-ying Xiang, Qi Chen, Xi-xi Tang, Lei Cao

**Affiliations:** 1 Department of Anesthesiology, Chongqing Cancer Institute, Chongqing University Cancer Hospital, Chongqing Cancer Hospital, 181# Hanyu Road, Shapingba District, 400030 Chongqing, China; 2 Present Address: Department of Anesthesiology, Xinqiao Hospital of Army Military Medical University, 83# Xinqiao Zhengjie, Shapingba District, 400037 Chongqing, China

## Abstract

**Objective:**

This meta-analysis compared the quality of lung collapse and the resultant adverse reactions between the use of double-lumen endotracheal tubes (DLT) and bronchial blockers (BB) in minimally invasive thoracic surgery.

**Methods:**

A search was performed in five bibliographic databases, namely PubMed, Springer, Medline, EMBASE, and Cochrane Library ignoring the original language, which identified five randomized controlled trials (RCTs) published on or before December 31, 2021. These studies were subsequently analyzed. All included studies compared the efficacy and safety of DLT and BB as a lung isolation technique in surgery. The methodological quality of each study was assessed by the Cochrane Collaboration’s risk of bias tool. The quality of lung collapse and the malposition rate were adopted as the main outcome indicators. Alternatively, the intubation time and the incidence of postoperative sore throat were adopted as secondary indicators.

**Results:**

When either DLT or BB were utilized in minimally invasive thoracic surgery, no differences were observed in the quality of lung collapse (odds ratio [OR], 1.00; 95% confidence interval [CI], 0.63 to 1.58), the intubation time (mean difference [MD], 0.06; 95% CI, -1.02 to 1.14), or the malposition rate (OR, 0.88; 95% CI, 0.37 to 2.06). However, the incidence of postoperative sore throat among patients treated with BB was significantly lower than that among patients treated with DLT (OR, 5.25; 95% CI, 2.55 to 10.75).

**Conclusion:**

When utilized in minimally invasive thoracic surgery, the quality of lung collapse with DLT was identical to that with BB. However, patients treated with the latter demonstrated a significantly lower incidence of postoperative sore throat.

## Introduction

Selective bronchial intubation was first used in practice in 1931 as a solution to thoracotomy-related pneumothorax [1]. Subsequent innovations in selective lung ventilation techniques have since greatly promoted the rapid development of thoracic surgery. In addition, the wide use of video technology, endoscopic instruments, and minimally invasive techniques has expanded the application of one-lung ventilation (OLV) from lung surgery to surgeries on the esophagus, heart, and other organs [2, 3].

At present, lung isolation remains the basis of thoracic anesthesia. In the past, the poor quality of atelectasis treatment could be compensated by manual extrusion and exposure. However, with the popularization of thoracoscopic surgery, the number of incisions in thoracic surgery has reduced from three to one, which, despite meeting the definition of minimally invasive surgery, poses higher requirements for atelectasis treatment. Existing clinical lung isolation techniques mainly use either DLT or BB. Multiple studies [4–6] have indicated that anesthesiologists prefer the former owing to the common belief that it provides a better quality of atelectasis treatment. However, neither the strong stimulation of double-lumen intubation nor the associated risk of postoperative sore throat can be ignored. Therefore, which technique is safer and more convenient in thoracoscopic surgery remains controversial.

The purpose of this study was to perform a systematic review and meta-analysis on RCTs comparing the applications of DLT and BB in thoracoscopic surgery, in order to identify differences in the quality of lung collapse, malposition rate, and incidence of postoperative sore throat between the two techniques.

### Methods

This meta-analysis followed the Preferred Reporting Items for Systematic Reviews and Meta-Analyses (PRISMA) guidelines [7] and was registered in PROSPERO on February 16th, 2022 (registration number: CRD42022302483). Subsequently, a systematic search was conducted among databases including PubMed, Springer, Medline, EMBASE, and Cochrane Library using keywords such as “double-lumen tube,” “bronchial blocker,” “lung isolation,” “one-lung ventilation,” and “thoracic surgery.” In addition, a list of included literature and related comments was finalized after reviewing the references. Only English literature was included in the study.

### Path diagram for inclusion of studies

**Fig. 1:**
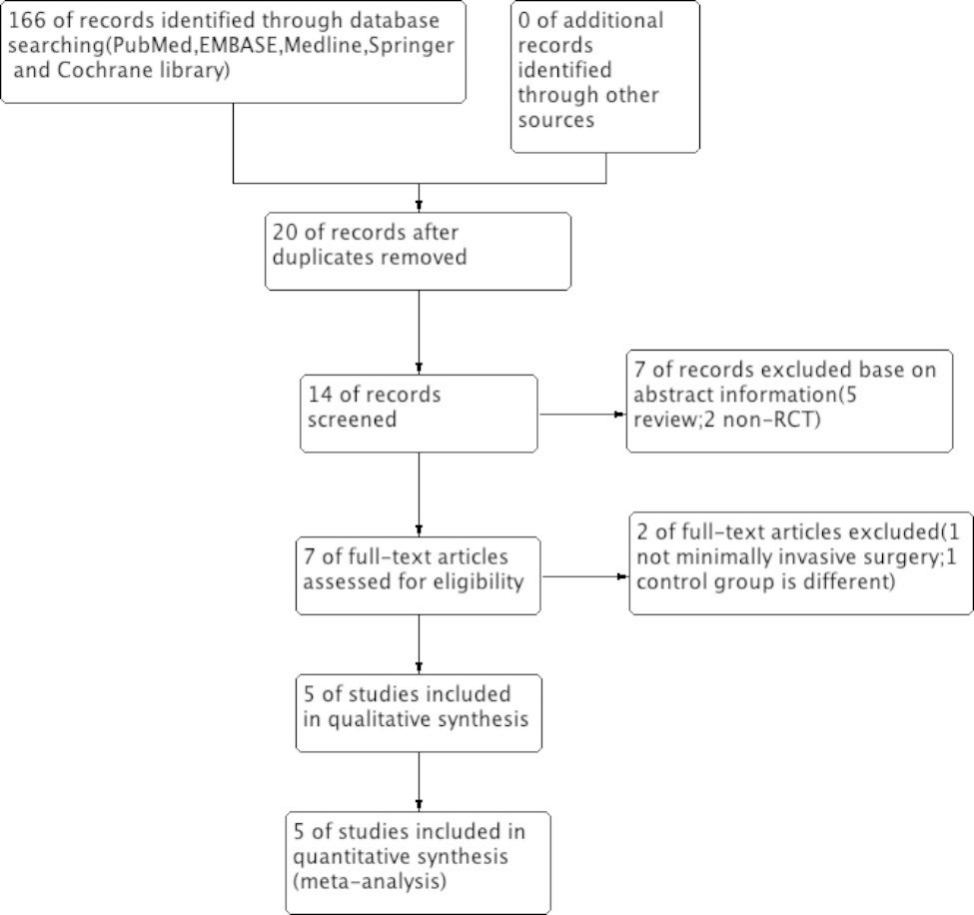
PRISMA flow diagram

Two authors (Y.Y.X and Q.C) independently determined the inclusion criteria based on the consensus between the two researchers. Conflicts were resolved by consulting the opinion of a third author (L.C). The quality of atelectasis treatment and the malposition rate were adopted as the main outcome indicators. The intubation time and the incidence of postoperative sore throat were adopted as secondary indicators. The results of the trial selection process are presented in the PRISMA flowchart (Fig. 1).

### Extraction of research characteristics and data

In addition to the names of the main authors, publication year, numbers of patients in each group, and types of catheters, this meta-analysis also collected data on the quality of dislocation rate, intubation time, malposition rate, and incidence of complications (Table 1). The data were extracted independently by two authors, and conflicts were resolved through reviews and discussions. Data on the mean value, standard deviation (SD), and the number of patients (n) were also extracted if they were reported or could be calculated.

**Table 1:** Characteristics of included studies

Author	Year	DLT/BB	Type of catheter(DLT/BB)	Gender	Outcome
BAUER [8]	2001	16/19	left-sided Broncho-Cath/ Wiruthan bronchial blocker	Both	The number of unsuccessful placement attempts, quality of lung collapse (excellent, fair, poor)
Bussie`res [9]	2016	20/18	Mallinckrodt™ left endobronchial tube/Fuji Uniblocker	Both	Time of lung collapse, quality of lung collapse (excellent, fair, poor)
Lu [10]	2017	21/19	Mallinckrodt Medical/Tappa Medical Technology	Both	Time of lung collapse, quality of lung collapse (excellent, fair, poor), complications (hypoxemia, sore throat)
Zhang [11]	2019	30/29	Mallinckrodt™ left endobronchial tube / Tappa Medical Technology	Both	The times of intubation and tube localization, lung collapse, complications (sore throat, hoarseness)
Zhang [12]	2020	28/27	Mallinckrodt Medical/ the Coopdech bronchial blocker	Both	Quality of lung collapse (excellent, fair, poor), complications (sore throat)

DLT: double-lumen endotracheal tube; BB: bronchial blocker

### Evaluation of research quality and risk of bias

**Fig. 2:**
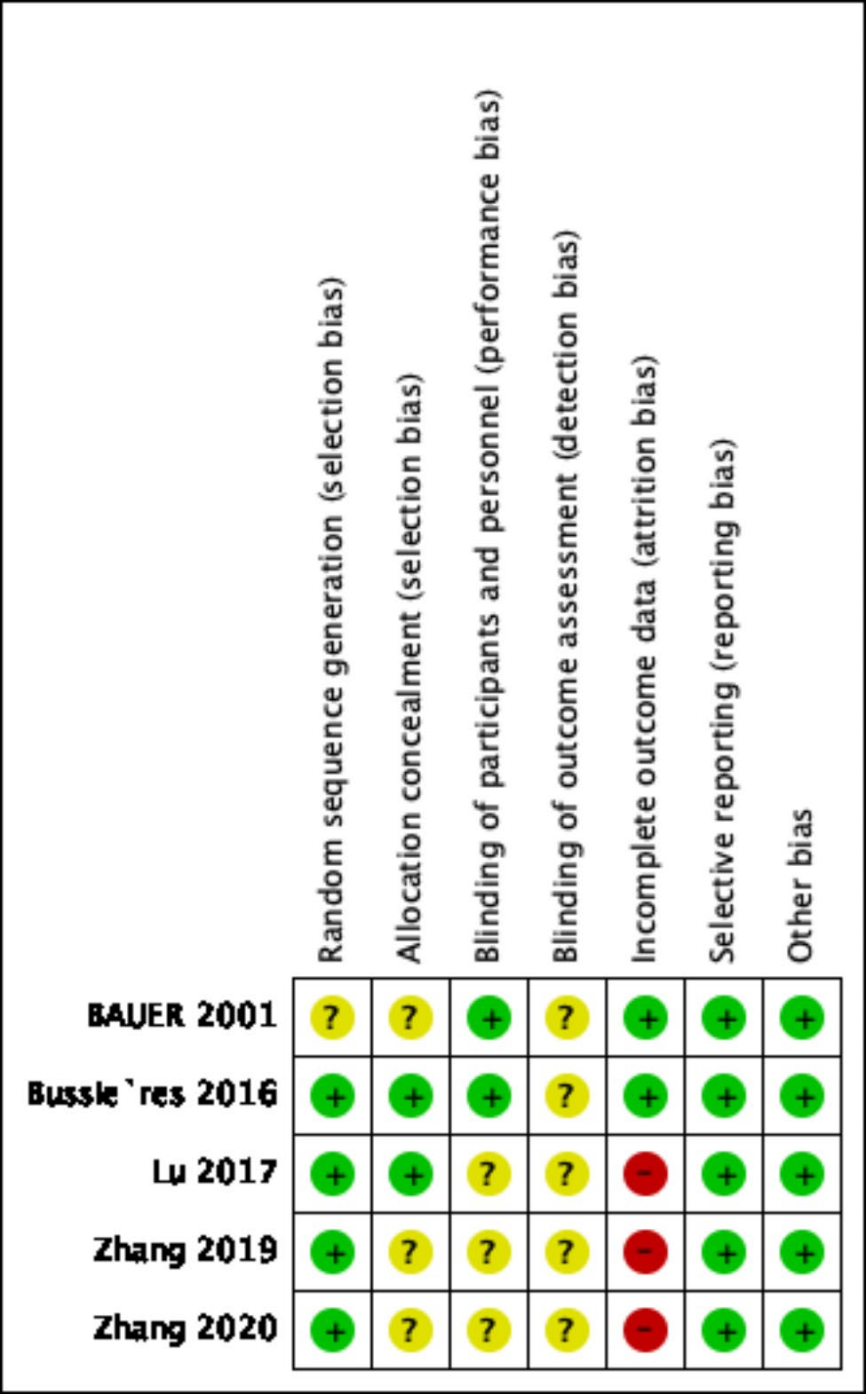
Evaluation of risk of bias for each included study. Green circle indicates low risk of bias, red circle indicates high risk of bias, yellow circle indicates unclear risk of bias

The quality of each RCT was assessed by two researchers (Y.Y.X and Q.C) using Cochrane Collaboration’s risk of bias tool. Various types of bias (selection, performance, detection, attribution, reporting, and other forms) were included in the assessment (Fig. 2). Subsequently, a quality score was generated for each RCT based on the consensus between the two authors. Conflicts were resolved by consulting the opinion of a third author (L.C). The quality score of the RCT was not considered the key factor for exclusion.

### Statistical analysis

All data analysis was performed using Review Manager 5.4 software (Cochrane Collaboration, Oxford, UK) and Stata 17 software (Stata Corp, College Station, TX, USA). Continuous data (intubation time) were expressed as mean deviations (MDs) or standard mean differences (SMDs) and 95% confidence intervals (CIs). Dichotomous data (quality of lung collapse, malposition rate, and incidence of postoperative sore throat) were expressed as odds ratios (ORs) and 95% CIs. The *χ*^*2*^ test (*P* value and *I*^*2*^ value) was used to evaluate heterogeneity. A random-effects model was selected in case of heterogeneity (P ≤ 0.05 or I^2^ > 50%) and a fixed-effects model was selected in case of homogeneity (P > 0.05 or I^2^ ≤ 50%).

## Results

### Literature selection and characteristics

A total of five 5 eligible RCTs were identified, involving 223 surgical patients. The characteristics of patients and the adopted intervention measures are listed in Table 1. All five RCTs compared the clinical safety of DLT and BB.

### Quality of lung collapse

**Fig. 3:**
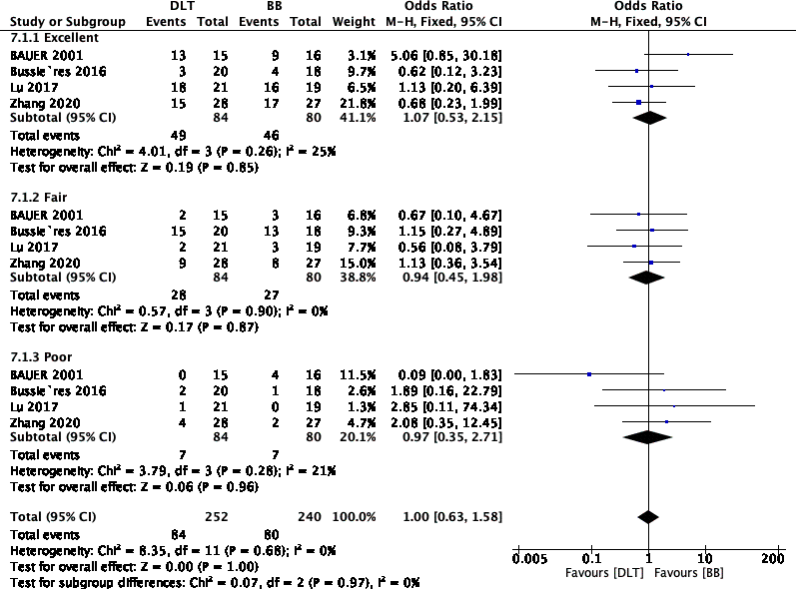
Forest plot showing the quality of lung collapse between DLT and BB.

Four RCTs (n = 164) reported the quality of lung collapse [8–10, 12], which was evaluated as either excellent, fair, or poor. There was no difference in the quality of lung collapse between the use of a DLT or BB in patients undergoing minimally invasive thoracic surgery (OR, 1.00; 95% CI, 0.63 to 1.58; I^2^ = 0%; P = 0.97) (Fig. 3). Implementing sensitivity analyses and Egger tests for publication bias did not significantly alter these results (P = 0.919).

### Device malposition rate

**Fig. 4:**
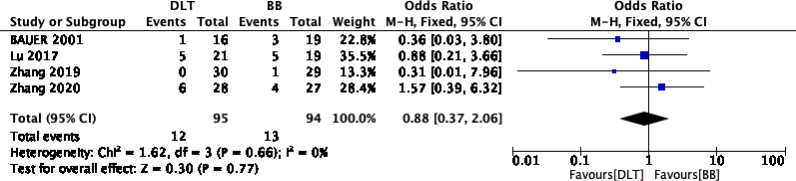
Forest plot showing the device malposition rate between DLT and BB.

Four RCTs (n = 189) recorded incidences of dislocation during lung isolation [8, 10–12]. No differences in the malposition rate were observed when either DLT or BB were utilized (OR, 0.88; 95% CI, 0.37 to 2.06; I^2^ = 0%; P = 0.77) (Fig. 4). Implementing sensitivity analyses and Egger tests for publication bias did not significantly alter these results (P = 0.133).

### Time for device placement

**Fig. 5:**
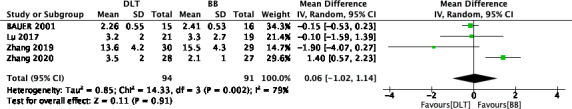
Forest plot showing the time for device placement between DLT and BB.

Four RCTs (n = 185) recorded the time required for placing the two devices [8, 10–12]. The time required to place the device in the correct position was not significantly different (RR, 0.06; 95% CI, -1.02 to 1.14; I^2^ = 79%; P = 0.91) (Fig. 5). Implementing sensitivity analyses and Egger tests for publication bias did not significantly alter these results (P = 0.978).

### Incidence of postoperative sore throat

**Fig. 6:**
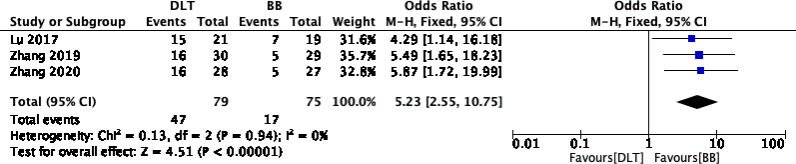
Forest plot showing the incidence of postoperative sore throat between DLT and BB.

**Fig. 7:**
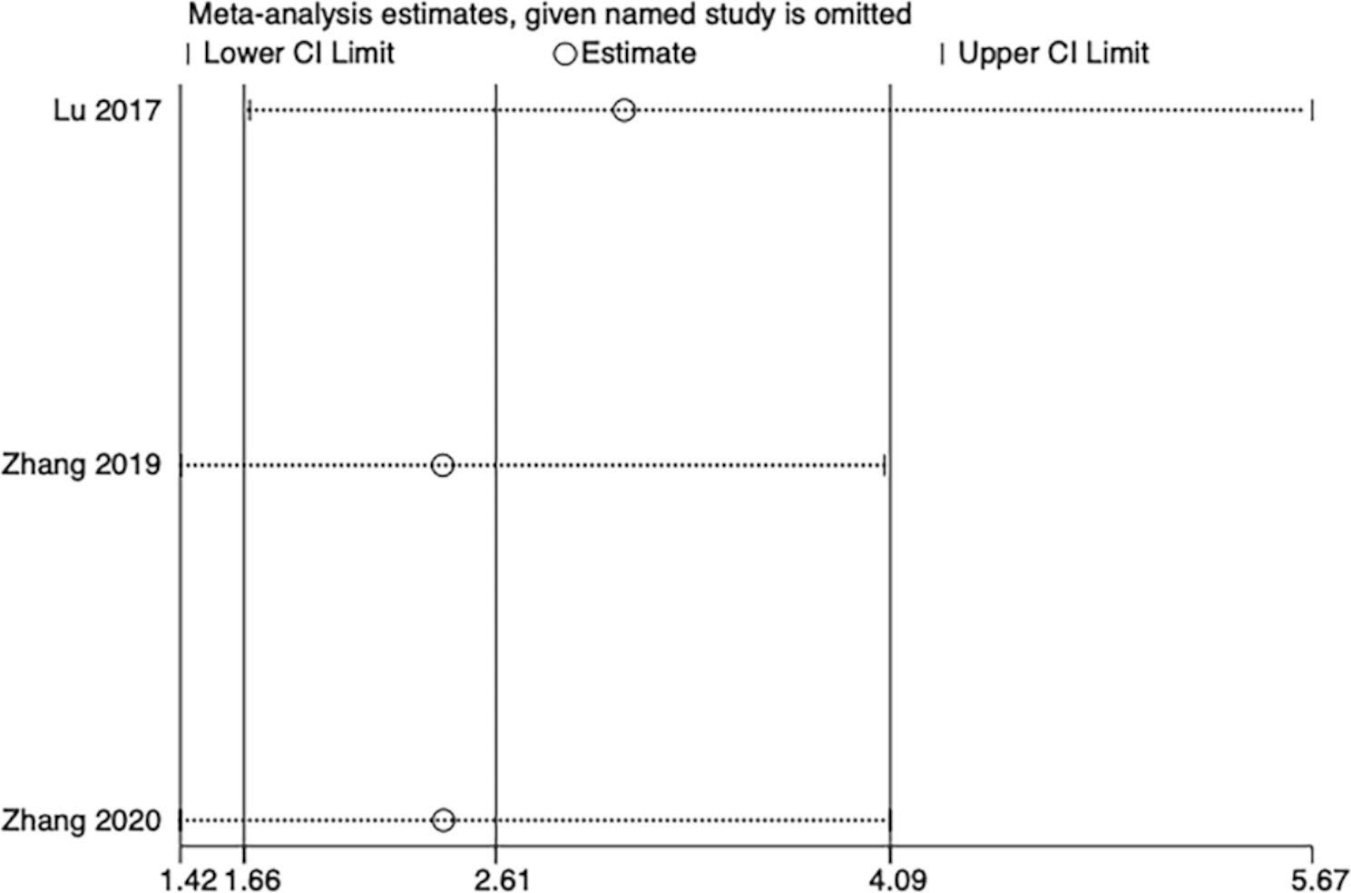
The plot of sensitivity analysis of the incidence of postoperative sore throat

Three RCTs (n = 154) recorded the incidence of postoperative sore throat [10–12]. The rate of postoperative sore throat was lower among patients treated with BB than among those treated with DLT (OR 5.23; 95% CI, 2.55 to 10.75; I^2^ = 0%; P < 0.00001) (Fig. 6). Implementing sensitivity analyses and Egger tests for publication bias did not significantly alter these results (P = 0.263). Implementing sensitivity analysis for the current meta-analysis was also performed, indicating that the results were reliable and statistically stable (Fig. 7).

## Discussion

This meta-analysis showed that while no differences were observed in the quality of lung collapse, device placement time, or malposition rate between BB and DLT, the incidence of postoperative sore throat with the former was lower than that with the latter.

During the anesthesia for minimally invasive thoracic surgery, lung isolation is mainly realized via BB and DLT [13, 14]. Some RCTs attempted to determine which of the two techniques was superior [15, 16]. A study suggested that the intubation time of BB was longer, while their malposition rate was higher [17]. An observational study that DLT provided better lung collapse and had a lower associated malposition rate, making it the safest choice. Left-DLT (L-DLT) was found to be the primary choice device for OLV also in the case of predicted or unpredicted difficult airways [18]. By contrast, another meta-analysis comparing the use of DLT and BB reported that despite a higher malposition rate, the operating time of BB was shorter [19]. Our study showed that there were no significant differences in either the intubation time or the malposition rate between DLT and BB.

Alternatively, the fact that DLT yields a higher risk of airway injury is well established [15, 20]. A study indicated that airway injury was most likely caused by the instrument used. Since DLTs have a larger diameter compared to BBs, they are more likely to damage the airway during intubation and extubation [20]. Another study suggested that airway damage was likely associated with operator skill [21]. Although a different study reported that the higher incidence of airway injury in the intubation of double-lumen endotracheal tubes was possibly related to their high dislocation rate [17]. A meta-analysis found that the incidences of postoperative hoarseness, sore throat, and air injury with double-lumen endotracheal tubes were substantially higher than those with bronchial blockers, indicating scope for improvement in double-lumen endotracheal tubes in the future.

For surgeries involving difficult airways or prolonged thoracic and esophageal surgeries, when postoperative respiratory support is needed, the fact that the endotracheal tube does not need to be replaced when BB are used reduces the risk of secondary airway injury and edema, which makes it superior to DLT. However, DLT play a crucial role in special operations such as pneumonectomy and bronchotomy [16].

When choosing a DLT or BB, a multidisciplinary intervention is necessary for the preoperative evaluation of the thoracic patient and whether to enter the ICU, with a full range of assessments, such as the patient’s gender, age, weight, cardiac function, lung function, and the need for postoperative organ support. These are all issues that need to be considered. In the perioperative multidisciplinary team, the anesthesiologist plays a leading role in optimizing the treatment strategy. We can choose DLT or BB according to different needs [22]. Some studies [23] still refer to DLT as the gold standard in various surgical procedures requiring lung isolations; moreover, it was preferred by most thoracic anesthesiologists. Considering the individual variation among patients, anesthesiologists should be proficient in using both DLT and BB.

This meta-analysis has some limitations. Most of the included literature compared the use of DLT and BB in the left lung, whereas few compared their uses in the right lung. This was likely related to the anatomy and ventilation requirement of the lung.

## Conclusion

This meta-analysis showed that there were no differences in the quality of lung collapse between DLT and BB in minimally invasive thoracic surgery. However, patients treated with DLT had a higher incidence of postoperative airway injury. Despite this, DLT still play an irreplaceable role in certain surgical procedures.

## Data Availability

The datasets generated and analyzed during the current study are available from the corresponding author upon reasonable request.

## Abbreviations

OR: Odds ratio
CI: Confidence interval
MD: Mean difference
RCT: randomized controlled trials
DLT: double-lumen endotracheal tubes
BB: bronchial blockers.
